# The Feet in Winter

**Published:** 1872-02

**Authors:** 


### THE FEET IN WINTER.

Through the feet comes every year to multitudes — death! Especially in winter time ought pains to be taken to keep the feet comfortably warm, to be healthful; the warmth comes from within. Stockings keep the feet warm, because they keep about the feet the warmth which comes from them; hence, those materials are best to clothe the feet which are the most perfect non-conductors; thus it is that woolen stockings are considered, as a general rule, "warmer" than cotton or silk; certainly there is less warmth in cotton or silk, because they convey away with rapidity what heat there may be in the feet. Yet some persons' feet are warmer, are more comfortable in winter with cotton stockings than with woolen, because persons with a vigorous circulation give out so much heat, that if kept about the feet, profuse perspiration is induced; this becomes condensed by the cold which comes through the boot or shoe, and the feet are thereby kept clammy and damp, and most disagreeably cold; but if such persons wear cotton or silk, the extra heat is conveyed away, still leaving enough to keep the feet comfortably warm.

If the pores of the skin are closed in any part of the body, that part is either unnaturally cold, or hot. If the circulation is vigorous there is over-heat, because it cannot get out of the body by these scape-pipes of the system. If the circulation is sluggish, if there is but little vitality, little life, the blood of the veins stagnates, and the blood of the arteries which carry heat and life cannot get there; hence the first step in keeping the feet warm is to keep the pores open. The pores of the soles of the feet are very much the largest in the whole body; hence the indispensable necessity of keeping these pores always open. That can be done in but one way: keep the feet clean; clean away the concretions of perspiration, and oil, and dust, which are always accumulating and which seal the tops of these pores, the little chimneys of the system, hermetically.

If the feet are not washed for a week or two in winter, and are then soaked in warm water for a quarter of an hour, a teaspoonful of a whitish substance can be scraped from the heel and centre of the soles with the finger-nails; this is dead skin, perspiration, dust, and other matters, all conglomerated, covering up the pores of the feet as effectually as if each had an iron hinge to lap over it; therefore soak the feet in warm water for at least a quarter of an hour every night before going to bed; twice a week scrape the soles well about the bottom of the heels with the finger-nails; nothing that can be named is half so efficiently safe; then dip both feet at a time in cold water for half a second, wipe with a towel and hold to a blazing fire until thoroughly dry, and get into bed without walking on a cold floor; the feeling of comfort after you have covered up will be an agreeable surprise. But before you dress in the morning put both feet in cold water for a second or two; they need not go deeper than to cover the toes; then wipe dry. This morning bath ought to be attended to every day in summer by all sedentary persons. Very many persons have got rid of the great discomfort of uniform cold feet in the way described. If persons have no health, no vitality, are not far from the grave, this as well as other methods will fail.

